# Erratum zu den Leitlinien für die molekulare und zytogenetische Diagnostik bei Prader-Willi-Syndrom und Angelman-Syndrom

**DOI:** 10.1515/medgen-2021-2060

**Published:** 2021-05-14

Wir möchten Sie darauf aufmerksam machen, dass bei den Leitlinien für die molekulare und zytogenetische Diagnostik bei Prader-Willi-Syndrom und Angelman-Syndrom, veröffentlicht in der medgen 2-2020, S. 169–176, leider nicht die finale Fassung, die von den Autoren eingereicht wurde, veröffentlicht wurde. Dadurch sind die letzten Korrekturen an der Leitlinie nicht mitaufgenommen worden. Die richtige Fassung dieser Leitlinie findet sich auf den Seiten der AWMF1https://www.awmf.org/leitlinien/detail/ll/078-010.html abrufbar.

Die wichtigste Korrektur bezieht sich auf die Textstelle im Kapitel 5.1. MS-MLPA.

Richtig heißt es dort nun:

*„Bei Patienten mit PWS wird eine komplette Methylierung der Sonden angezeigt (Hypermethylierung), entweder da das unmethylierte **paternale** Allel deletiert ist oder weil bei einer maternalen UPD oder einem Imprintingdefekt beide Allele methyliert vorliegen.*

*Bei AS hingegen zeigen die Sonden meist keine Methylierung (Hypomethylierung) entweder aufgrund der Deletion des methylierten **maternalen** Allels oder aufgrund einer paternalen UPD oder Imprintingdefektes, bei denen beide Allele unmethyliert vorliegen.“*

